# Limiting Damage during Infection: Lessons from Infection Tolerance for Novel Therapeutics

**DOI:** 10.1371/journal.pbio.1001769

**Published:** 2014-01-21

**Authors:** Pedro F. Vale, Andy Fenton, Sam P. Brown

**Affiliations:** 1 Centre for Immunity, Infection, and Evolution, School of Biological Sciences, University of Edinburgh, Edinburgh, United Kingdom; 2 Institute of Evolutionary Biology, School of Biological Sciences, University of Edinburgh, Edinburgh, United Kingdom; 3 Institute of Integrative Biology, University of Liverpool, Liverpool, United Kingdom

## Abstract

In the field of infectious disease control, novel therapies are focusing on reducing illness caused by pathogens rather than on reducing the pathogen burden itself. Here, Vale and colleagues highlight some potential consequences of such therapeutics for pathogen spread and evolution.

## Summary

The distinction between pathogen elimination and damage limitation during infection is beginning to change perspectives on infectious disease control, and has recently led to the development of novel therapies that focus on reducing the illness caused by pathogens (“damage limitation”) rather than reducing pathogen burdens directly (“pathogen elimination”). While beneficial at the individual host level, the population consequences of these interventions remain unclear. To address this issue, we present a simple conceptual framework for damage limitation during infection that distinguishes between therapies that are either host-centric (pro-tolerance) or pathogen-centric (anti-virulence). We then draw on recent developments from the evolutionary ecology of disease tolerance to highlight some potential epidemiological and evolutionary responses of pathogens to medical interventions that target the symptoms of infection. Just as pathogens are known to evolve in response to antimicrobial and vaccination therapies, we caution that claims of “evolution-proof” anti-virulence interventions may be premature, and further, that in infections where virulence and transmission are linked, reducing illness without reducing pathogen burden could have non-trivial epidemiological and evolutionary consequences that require careful examination.

## Two Ways of Surviving Infection

**Figure 1:**
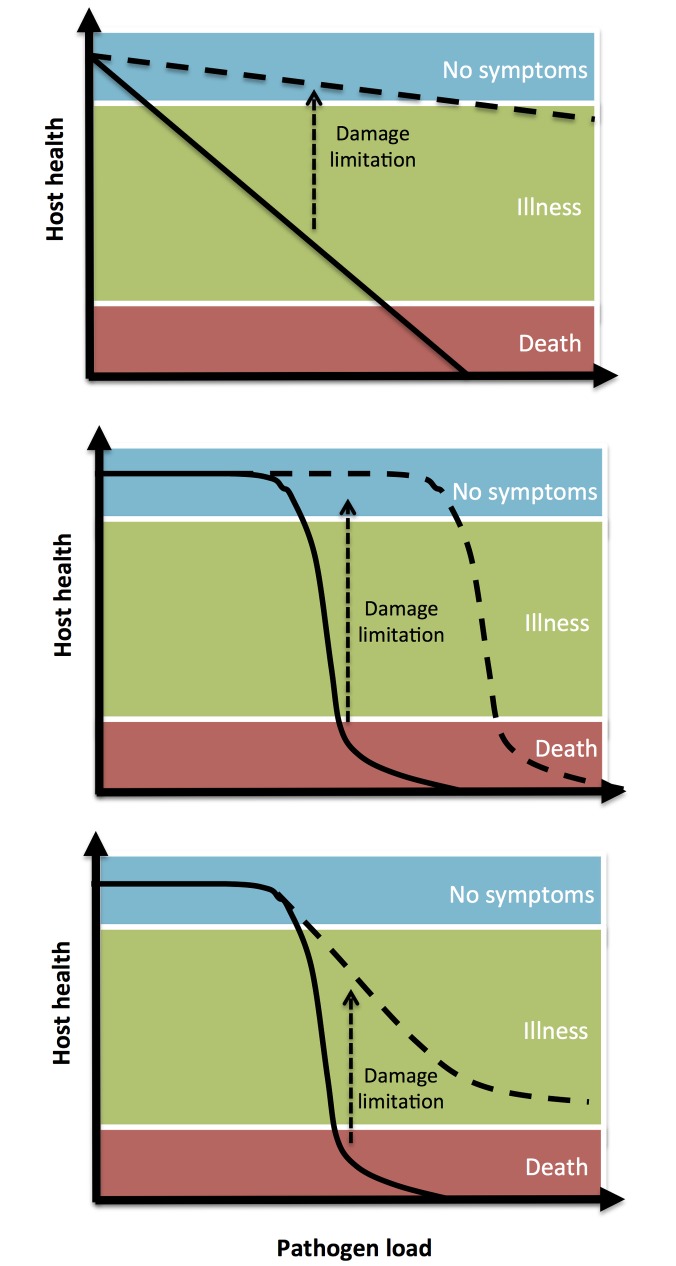
The effect of damage limitation mechanisms on the loss of host health during infection. See Box 1 for further details.

When organisms become infected, there are two ways to minimize virulence (here defined as damage leading to morbidity or mortality). One way is to eliminate pathogens directly. An additional way is using mechanisms that, while not reducing pathogen loads directly, reduce the damage caused by their growth (Box 1; Figure 1) [1]–[3]. Treating infectious disease has often taken the road of pathogen elimination, either by administering antimicrobial drugs or by stimulating host immune responses with vaccination to achieve the same goal. There are, however, demonstrated drawbacks to pathogen elimination [4]–[8]. Notably, one unintentional and very undesirable side-effect of interventions that kill pathogens is that they impose strong selection for faster growing, and/or more resistant pathogens; when elimination therapies are imperfect or incomplete, they also leave behind the few pathogens that are the most capable of avoiding them [4]–[8].

**Box 1. The Effect of Damage Limitation Mechanisms on Host Disease Tolerance**

As pathogen loads increase during infection, hosts will lose health, going from a state of no symptoms to illness and, in extreme cases, death (Figure 1). Hosts with more efficient damage limitation are able to maintain a higher level of health during infection. These hosts are able to sustain higher pathogen loads but experience a less severe decline in health than less tolerant hosts. One can imagine several relationships between increasing pathogen load and host health, which may be infection- or pathogen-specific (Figure 1). Theory has highlighted how the nature of these specific relationships are important in determining how pathogens evolve and spread when host disease tolerance increases [24]. While boosting disease tolerance is generally predicted to lead to an increase in prevalence, the rate at which pathogens evolve to grow and harm their hosts can either increase or decrease depending on the shape of the relationship between host health and pathogen load [24],[30]. The curves drawn in Figure 1 represent the level of health experienced by a population of hosts for a given pathogen load, in the presence or absence of damage limitation treatments [24]. To fully grasp the dynamic nature of damage limitation during infection it is important to take repeated measures of host health matched for pathogen loads. Plotting the time-ordered behaviour of individual host health and pathogen loads has been proposed as a useful method of describing a range of alternative trajectories from illness back to health, which could be useful to identify options for personalized anti-infection treatments [11],[57].

**Figure 2:**
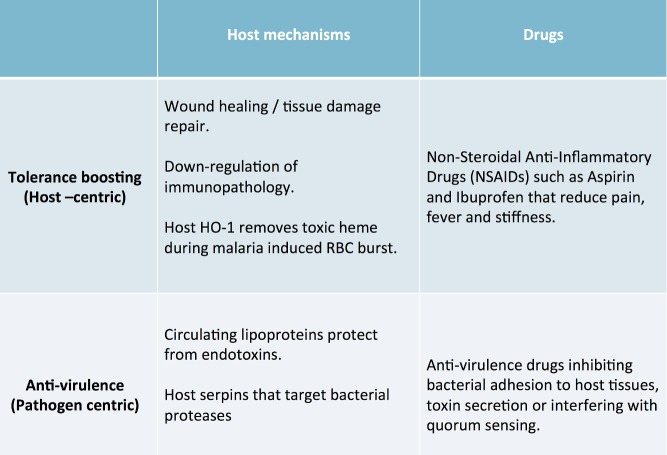
A simple framework for damage limitation. Damage limitation host mechanisms or drugs may be either host-centric, improving the host's capacity to tolerate infection, or pathogen-centric, targeting pathogen derived molecules that promote virulence. In all cases, damage limitation improves host health without directly eliminating pathogens. See Box 2 for further details.

To circumvent the drawbacks of pathogen elimination, and generate more sustainable treatments of infection, an increasingly popular view is to focus less on pathogen control and more on damage limitation during infection [9]–[12]. Instead of eliminating pathogens, novel therapeutics are focusing on alternative ways of disarming pathogens, such as interfering with quorum-sensing and secretion systems, inhibiting toxin production and diffusion, and limiting the efficiency of bacterial adhesion mechanisms (Box 2; Figure 2; also see Table 1 in [13]). One particular motivation for this suggestion is the belief that, by not targeting the pathogen directly, these approaches will not select for pathogen resistance strategies (as is seen in the case of conventional drugs) or increased pathogen virulence [13]–[15]. While this change in direction seems promising, the truth is we know very little regarding the potential consequences of damage limitation therapies for pathogen spread and evolution in the long run. It may be prudent to learn from history, as once “fool-proof” strategies such as antibiotics and vaccines have also been accompanied by the undesirable outcomes of multidrug resistant bacteria [16]–[18] and vaccine escape variants [7],[8]. However, we may be able to borrow concepts and approaches from disease evolutionary ecology, much of which have been developed in the light of conventional drug resistance and virulence evolution [19], to predict likely responses to damage limitation therapies. Below, we outline a simple framework for considering the epidemiological and evolutionary consequences of damage limitation during infection. We highlight the important distinction that damage limitation, be it via therapeutic drugs or mechanisms that hosts have evolved, may be either host- or pathogen-centric. We then discuss how this distinction is useful in understanding some potential consequences that damage limitation interventions may have for both the spread of the disease and the evolution of virulence.

**Box 2. Mechanisms of Damage Limitation**

***Host anti-virulence.*** Some host mechanisms promote milder disease by eliminating pathogen-derived toxins. For example, it has been shown that cytokine-induced increases in physiological levels of serum lipids may protect animals from lipopolysaccharide (LPS) toxicity during septic bacterial infection [58],[59]. Host serpins have also been found to inhibit bacterial cysteine-proteases, thereby protecting the host from the virulent effect of infection, without eliminating infection altogether [60]. These host mechanisms reduce the damage caused during infection by targeting pathogen-derived toxins (that is, they target virulence) without directly reducing the number of pathogens, resulting in increased host damage limitation (Figure 2).

***Host disease tolerance.*** Other host mechanisms promote tolerance during infection by promoting improved host condition. For example, host heme oxygenase 1 (HO-1) degrades toxic heme released in the burst of red blood cells during malaria infections [61]–[63]. Similarly, the Th2 response has recently been found to reduce pathogenesis during helminthic infection by both recruiting macrophages to repair tissue damage, and by down-regulating inflammation [64]. These examples underline how the sources of disease may come from both the infection, and from the host response to the infection [21]. Mechanisms that help to regulate this response efficiently to reduce immunopathology also follow the functional definition of disease tolerance, because they increase host health during infection in a way that is independent of pathogen loads (Figure 2) [51].

***Pro-tolerance drugs.*** Beyond host mechanisms, some medical interventions also improve host health without eliminating pathogens. Common examples include non-steroidal anti-inflammatory drugs (NSAIDs) such as aspirin and ibuprofen, which are typically used to reduce pain, inflammation, and stiffness, but do not directly reduce infection burdens (Figure 2) [65].

***Anti-virulence drugs.*** A novel class of drugs that has been proposed to promote damage limitation by interfering with the causes of virulence without eliminating pathogens [13]–[15]. Anti-virulence drugs are a class of compounds that are neither bacteriocidal nor bacteriostatic, but rather reduce virulence by inhibition of bacterial adhesion to host tissues, inhibiting the secretion of bacterial toxins, or by interfering with the quorum-sensing signalling between bacteria that frequently modulates virulence factor expression [33],[46],[47],[60],[66]–[72]. Anti-virulence drugs therefore reduce pathogenesis but pathogen loads are not directly targeted. While this intervention is clearly pathogen-centric (Figure 2), the outcome is analogous to host disease tolerance because hosts are able to maintain health despite harbouring high infection burdens.

## How Can We Limit Damage during Infection?

Just as hosts have evolved resistance mechanisms that identify and eliminate pathogens during infection [20], alternative natural mechanisms that promote damage limitation also exist, and may act either on the host or on the pathogen (Box 2; Figure 2). These may be classified into mechanisms that improve host condition during infection, such that hosts become more tolerant of infection (“pro-tolerance” mechanisms) [1]–[3],[21], or alternatively mechanisms that target pathogen-derived toxins, or that interfere with pathogen signalling (but importantly that do not eliminate pathogens), which have been termed “anti-virulence” mechanisms [10],[14],[15]. Given the relative success in harnessing the power of the immune system for pathogen elimination (e.g., vaccination), a logical question is whether we can equally replicate the damage limitation mechanisms that hosts have evolved to reduce the severity of infectious disease. Many commonly used anti-inflammatory drugs already follow this basic principle (Box 2; Figure 2), because they focus on alleviating the symptoms of infection without directly eliminating its cause. More recently, a novel class of “anti-virulence” drugs has also emerged [13]–[15], that reduce pathogenesis by targeting bacterial compounds without actually eliminating pathogens directly (Box 2; Figure 2). These new approaches seem attractive. First, from the perspective of the patient, reducing illness, whether or not killing the cause, is always the main priority. Second, by not targeting pathogen growth directly, mechanisms that promote damage limitation have been proposed to reduce selection for faster growing pathogens, and in principle temper the evolution of drug resistant or vaccine escape variants. However, as we discuss below, unlike pathogen elimination therapies, the epidemiological and evolutionary consequences of damage limitation interventions are currently poorly understood and deserve careful attention.

## What Are the Consequences of Damage Limitation for Disease Spread?

**Figure 3:**
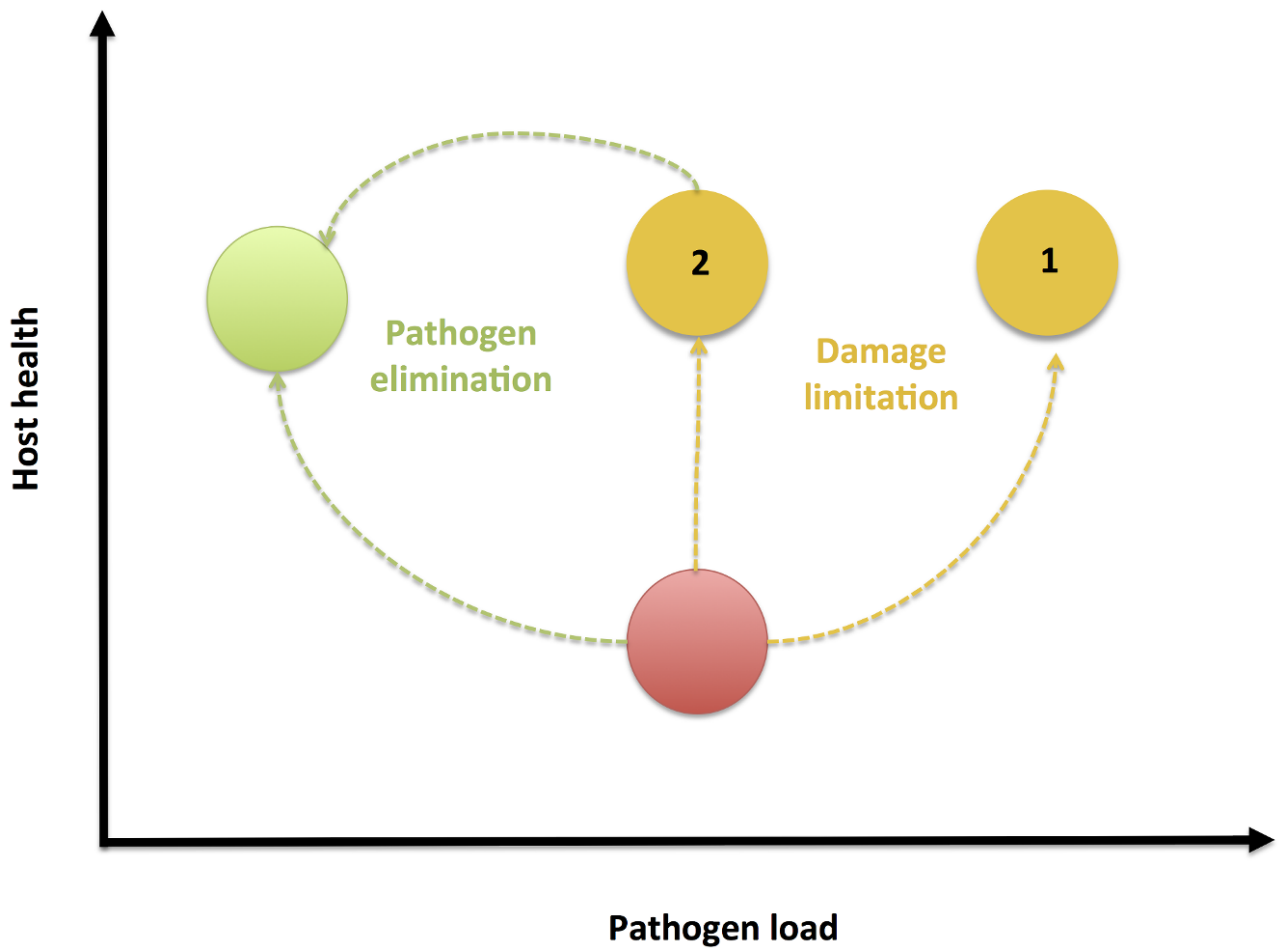
Two roads to health: elimination and damage limitation. During infection, pathogen growth causes tissue damage that reduces host health (red circle). Health may be regained through mechanisms that eliminate pathogens (green circle), or instead by mechanisms that reduce the damage caused by pathogens (yellow circle). Such damage limitation mechanisms improve health without reducing pathogen burdens, and therefore could result in hosts being able to tolerate even higher pathogen burdens (scenario 1). Alternatively, reducing the damage caused by infection could also allow the host immune response to eliminate these pathogens (scenario 2). It is currently unclear how host mechanisms of pathogen elimination interact with damage limitation mechanisms.

The evolutionary ecology of host disease tolerance has received significant study [22]–[32]. This work can therefore offer valuable insight into the epidemiological and evolutionary consequences of therapeutics that focus on damage limitation instead of pathogen elimination, at least for drugs that promote host disease tolerance. By definition, tolerant hosts are able to maintain relatively higher health as pathogen loads increase during infection (Box 1; Figure 1) [1]–[3]. Furthermore, there may be additional benefits in improving damage limitation: it has been proposed that by reducing pathogenesis during infection, anti-virulence drugs could buy the immune system valuable time to clear infection [14],[15],[33], thereby leading to increased pathogen elimination indirectly. The conditions under which this may occur are currently unclear, and so understanding the interplay between anti-virulence drugs and host immunity remains an important question to be addressed. For example, mice experimentally infected with *Mycobacterium tuberculosis* and treated exclusively with the common anti-inflammatory drug ibuprofen showed reduced lung lesions, and increased bacterial clearance and survival relative to control mice, presumably because of a reduction in inflammation-related immunopathology [34]. However, a possible adverse consequence of treatments that improve host health without eliminating pathogens is that hosts will present higher pathogen loads, which may result in more opportunities for transmission (Figure 3) [35]. For example, mice with a deficient cyclooxygenase (COX) pathway, mimicking the effect of COX-2 inhibitor anti-inflammatory drugs, infected with influenza A showed less severe disease symptoms but had markedly higher lung viral titres [36]. A similar outcome was found in murine *Trypanosoma cruzi* infection, where treating with a variety of anti-inflammatory cyclooxygenase inhibitors increased mouse health, but also increased parasitaemia relative to control mice [37]. Earlier work in double-blind clinical trials also found that treating human rhinovirus infections with aspirin resulted in elevated viral shedding rates despite reducing the severity of other disease symptoms [38].

**Figure 4:**
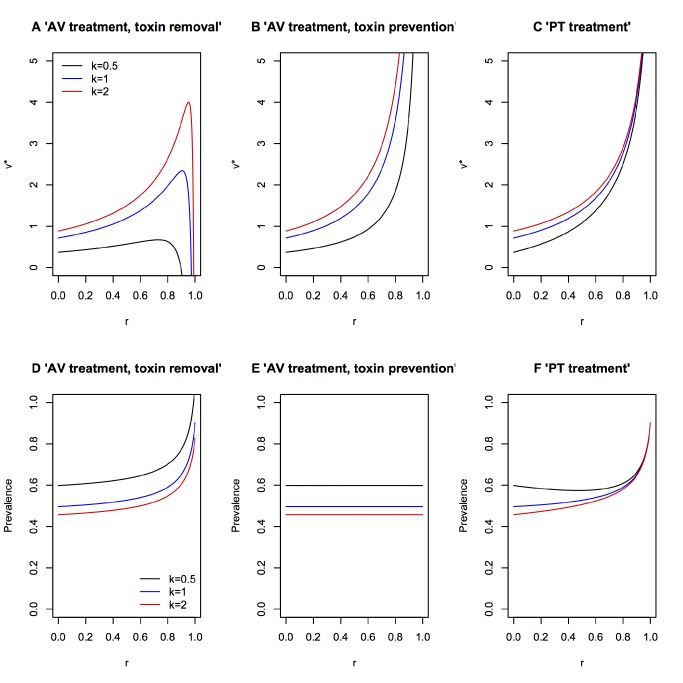
Epidemiological and evolutionary consequences of damage limitation treatments. Evolutionarily stable virulence factor production v* (A–C) and prevalence (D–F), plotted against anti-virulence (AV) or pro-tolerance (PT) drug efficacy, r. We plot different levels of how virulence and transmission are related (*k*). Model details are described in Box 3. Parameter values are as in [56] *b1* = 1, *b2* = 0.5, *d* = 1, *a* = 0.2, *σ* = 0.1, *c* = 0.05. Varying the cost of virulence factor production *c* does not affect the overall trend of the results, although it does affect the magnitude v* and prevalence.

As a consequence of reducing the severity of the symptoms of infection, hosts may therefore show increased potential for transmission (Figure 3) [1],[24],[30]. This may be problematic for two reasons. First, while treated hosts may be able to tolerate disease, increased transmission means there is obvious danger for less tolerant neighbouring populations or migrant hosts that have not benefitted from the damage limitation treatment [30]. Second, in the absence of clear disease symptoms, detection of infection may be compromised [39], which could further aid disease spread to less tolerant hosts. In Box 3 and Figure 4 we show that the overall effect of damage limitation therapies on the prevalence of infection depends on whether anti-virulence drugs target the production of pathogen virulence factors or simply alleviate their effects (Box 3; Figure 4E and 4F). For both anti-virulence and pro-tolerance classes of damage limitation therapies however, there is a real possibility of increasing the overall prevalence of infection, particularly for high efficacy drugs (Box 3; Figure 4). This scenario may be less problematic when the symptoms being targeted are directly responsible for transmission (as in the case of sneezing and coughing) or if they are the consequence of an opportunistic or accidental infection, and therefore present a dead-end to disease transmission [40]. For other types of infection however, damage limitation therapies may have less desirable population-level effects, which must be carefully considered.

**Box 3. Pathogen Evolution in Response to Damage Limitation Therapies**

To illustrate how anti-virulence (AV) or pro-tolerance (PT) therapies (Box 2; Figure 2) may affect the evolution of pathogen virulence we modify the framework presented by Gandon and colleagues [56], which considered the optimal level of pathogen production of a potentially costly toxin virulence factor (VF) under anti-toxin vaccination. To understand the effect of these damage limitation therapies on both pathogen evolution and disease spread, we modified this model to incorporate a weighting factor (*k*), which determines how much the VF production contributes to transmission:
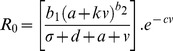
where *R*_0_ is the pathogen's basic reproduction number, a proxy for pathogen fitness; *b_1_* is the pathogen baseline transmission rate; *b_2_* is the exponent of transmission function; *d* is the baseline host mortality rate; σ is the pathogen clearance rate; *a* is the baseline (non-toxin) pathogen virulence due to within-host growth; *v* is the rate of toxin production; and *c* is the cost of toxin production. Here we use this simple framework to illustrate how anti-virulence and pro-tolerance damage limitation therapies influence the pathogen's optimal rate of VF production, v*, assuming that evolution acts to maximise *R*_0_.

**Anti-virulence drugs**

We first consider the effect of anti-virulence (AV) drugs that act directly on the toxin virulence factor, reducing its supply, with efficacy *r*. We call this model the “AV toxin removal” model:
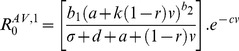
Note that under AV drug treatment hosts may still suffer from infection due to pathogen growth (*a*), because AV drugs only target the virulence factor (VF) component of virulence (*v*). The above equation assumes the pathogen still makes the toxin and incurs the cost of producing it, although the toxin is not effective. An alternative may be that the AV drug stops toxin production. In this case, the AV drug reduces pathogenesis, but also has the collateral effect of alleviating the cost to the pathogen of producing the toxin. We call this model the “AV toxin prevention” model:
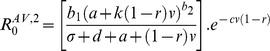

**Pro-tolerance drugs**

We now consider the effect of pro-tolerance (PT) drugs, which are drugs that alleviate the severity of disease by targeting host damage without directly affecting pathogen growth rate (Box 2; Figure 2). PT drugs therefore act on the overall damage caused by the pathogen (*a*+*v*), leading to:
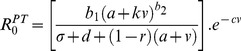

**Evolution of toxin production under the different damage limitation therapies**

The specific mechanism of drug action can have a major impact on the outcome of pathogen evolution: if AV drugs render toxins ineffective but leave the costs of toxin synthesis intact (“AV toxin removal” model; Figure 4A), increasing AV drug efficacy tends to select for increased toxin production, but will drive toxin production down at very high drug efficacies (to reduce redundant investment costs), particularly if the link between toxin production and transmission (*k*) is low. By contrast, if AV drugs act by stopping toxin production (“AV toxin prevention” model; Figure 4B), increasing drug efficacy always selects for pathogen strains that increase their intrinsic rate of toxin production. PT drugs have a similar effect on pathogen evolution, always selecting for high toxin production as drug efficacy increases (Figure 4C).

In all scenarios the quantitative level of virulence factor (VF) production depends on how it is related to transmission. When the disease symptoms that arise from VF production are weakly related to transmission (low *k* values), the optimal level of toxin production is lower than when it is strongly linked to transmission, but the effect of *k* is less important for the PT therapies (Figure 4C) than the other scenarios explored. Furthermore, very low *k* values can prevent the escalation of virulence seen under the AV toxin removal model (Figure 4A), as the continued cost of virulence factor production coupled with a weak transmission benefit leads to virulence factor investments becoming futile.

In terms of selection for virulence, damage limitation therapies would therefore appear to be more attractive options for the relief of symptoms that are not directly linked to transmission (low *k*), compared to symptoms like sneezing or coughing, that act as catalysts for disease spread. Alternatively, the use of AV drugs that are highly efficient at inactivating toxins (high *r*) may be an option, provided pathogens still pay a fitness cost for producing them (Figure 4A and 4B). What is clear is that the selective effects of damage limitation therapies, regardless of whether they target the host or the pathogen have non-negligible effects on pathogen evolution.

**Consequences of pathogen evolution and drug therapies for pathogen prevalence**

We now explore the population-level epidemiological consequences (infection prevalence) arising from the combination of the above drug therapies and the consequent evolved virulence levels. To do this we used the following population dynamic framework to explore how equilibrium pathogen prevalence (P*) varies with efficacy of each drug:
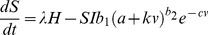


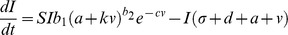
where *S* and *I* are the numbers of susceptible and infected hosts respectively, *H* ( = *S*+*I*) is the total host density and λ is the per capita host growth rate; all other parameters are as defined above. From these equations, the equilibrium pathogen prevalence is given as:
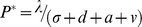
We can then modify this framework to incorporate the different treatment scenarios described above, calculating the appropriate optimal virulence in each case, and the subsequent consequences for P*. For both the AV toxin removal (Figure 4D) and PT (Figure 4F) models, increasing drug efficacy initially has little effect on overall infection prevalence of infection but, at very high drug efficacies, will tend to drive infection prevalence upwards. The population-level prevalence of infection under the AV toxin prevention drug (Figure 4E) is unaffected by drug efficacy. Overall, combining the results from the evolutionary and epidemiological analyses suggests that (i) highly effective AV toxin removal drugs can lead to the evolution of highly prevalent but relatively benign pathogens (Figure 4A and 4D), (ii) highly effective AV toxin prevention drugs can select for highly pathogenic pathogens of intermediate prevalence (Figure 4B and 4E), and (iii) highly effective PT drugs provide arguably the worst-case scenario, potentially selecting for highly prevalent and highly virulent pathogens (Figure 4C and 4F).

## What Are the Consequences of Damage Limitation for Pathogen Evolution?

There are two main reasons why we should care about the effects of damage limitation on pathogen evolution. One is the potential for pathogens to evolve resistance to drugs and vaccines; the other is the potential for the evolution of pathogens that cause more virulent infections. At first glance, the potential effects of damage limitation on pathogen evolution might appear desirable: pathogens are not eliminated, so selection for increased within-host growth rates is reduced, and therefore the scope of pathogen evolution under damage limitation therapies has been proposed to be limited [10],[14],[15].

However, there is currently little evidence to support this suggestion, and strong reasons to expect the opposite outcome. Pro-tolerance and anti-virulence treatment strategies have a potentially important difference with regard to the risks of drug resistance. While they do not eliminate pathogens, anti-virulence drugs directly target pathogen phenotypes, and so present ample opportunities for direct evolutionary responses to restore a pathogen phenotype that potentially increases its fitness [41]. For example, simple changes to pathogen efflux pump regulation have been shown to restore virulence expression in the face of anti-virulence drug treatments [42]. In contrast, we may expect the host-centric nature of pro-tolerance drugs to significantly reduce the scope for selection of resistance mechanisms in pathogens. However, by changing the level of damage experienced by an infected host they could still affect the evolution of virulence. This may be especially true of infections where virulence and transmission are linked, via a trade-off [43]: high levels of transmission require high levels of within-host growth, but this may also kill the host prematurely, resulting in overall lower pathogen transmission. High virulence therefore presents a cost to the pathogen, whose fitness is expected to be maximized instead at intermediate levels of virulence (see [6],[44],[45] for empirical examples of this trade-off). Pro-tolerance drugs would alleviate this cost, because pathogens can exploit hosts more without the risk of host death becoming too severe. Under this scenario, it is possible that pro-tolerance interventions could result in more virulent pathogens. Indeed, our evolutionary analysis (Box 3) suggests that selection for increased virulence is a possible outcome of both anti-virulence and pro-tolerance damage limitation treatments (Box 3; Figure 4A–4C).

Previous theoretical work dealing with pathogen evolution under variable levels of disease tolerance suggest that both low and high virulence evolution is possible depending on exactly how pathogen within-host growth impacts host health [24],[30]. Empirical tests of these predictions are scarce, but where available, they suggest caution in how damage limitation therapies are applied. For example, quorum-sensing (QS) signalling in bacteria, which commonly induces the expression of virulence factors, is one of the main targets of current anti-virulence drugs [46]–[48]. A recent study in intubated patients showed that inhibiting the QS signalling of *Pseudomonas aeruginosa* relaxed selection for less virulent loss-of-function mutants, resulting in the evolution of more virulent strains during the course of infection [49]. The reason for this is that QS loss-of-function mutants are social cheats; they use the QS signal produced by the wild-type strain, but do not pay the cost of producing it. QS loss-of-function mutants are also less virulent than the wild type. In the absence of an anti-virulence drug that blocks QS signalling (in this case, azithromycin), these cheating mutants increased in frequency over time because they don't pay the fitness cost of QS signalling. Adding azithromycin significantly reduced QS-gene expression, and any advantage of social cheating was lost. As a result of adding an anti-virulence drug, selection for lower-virulence mutants was therefore relaxed, and the more virulent wild-type isolates increased in frequency.

Interventions that attempt to limit damage by inhibiting quorum-sensing dependent virulence factor production (similar to the “toxin prevention” situation we model in Box 3) could therefore, in principle, select for higher virulence. This outcome is by no means certain and applies most for infections where pathogen growth and virulence are highly linked to transmission; if they are only weakly linked to transmission (low *k* in Figure 4) then toxin removal anti-virulence drugs can select for reduced virulence (Box 3; Figure 4A). In other types of infection, for example opportunistic or accidental pathogens [40], or when virulence is mainly the consequence of an over exuberant immune response [12],[50],[51], the trade-off hypothesis of virulence evolution will not apply, and damage limitation therapies may be very promising in reducing disease severity without the concern for the effects on disease transmission or evolution. Generally however, we currently lack adequate experimental data to confidently predict the effect of damage limitation treatments on pathogen evolution.

## Perspectives and Outstanding Questions

Damage limitation presents a promising alternative to pathogen elimination, and in some types of infection might be useful in reducing disease symptoms while aiding immune clearance. Certainly, it is important to put these treatments in context with the available alternatives. Treating patients is an imperative, yet among the clinically equivalent options, we may wish to choose the one that minimizes the risks of drug resistance and virulence evolution. Currently, regarding anti-virulence drugs and other damage limitation therapies there are important population-level consequences that remain to be fully understood. We focused on the distinction between pro-tolerance (host-centric) and anti-virulence (pathogen-centric) therapies, and we found that this distinction may be important when considering the epidemiological and evolutionary consequences of damage limitation interventions (Box 3). In practice, microbial evolution is rapid and inescapable, and a more fruitful approach may be to use evolution to aid the development of better treatments [19]. For example, it would be especially useful to test the feasibility of damage limitation treatments that specifically select for reduced virulence and resistance, such as anti-virulence drugs that alleviate symptoms that are not strongly linked to transmission (Box 3; Figure 4A). Here it may be useful to expand our knowledge of damage limitation mechanisms from mammals to other organisms that may reveal novel mechanisms for damage limitation therapeutics [52]. Beyond therapeutic measures we would also benefit from a better understanding of the host genetic control of damage limitation mechanisms (Box 2; Figure 2) [3],[10]. For example, a better knowledge of pro-tolerance and anti-virulence mechanisms may help guide livestock genetic improvement programs, preventing the use of chemicals in the future [53]–[55]. Theoretical predictions of pathogen evolution under varying levels of disease tolerance are also limited [24],[30],[56], and future models could be tailored to understand specific types of host-pathogen interactions. A critical question that is currently unclear is how damage limitation drugs might interact with the immune system: will they simply maintain host health, buying time to mount a stronger and more efficient immune response [33]? Or will tolerant hosts become symptomless, potentially dangerous carriers of disease [10],[30]? The answer to these questions will be crucial to the long-term success or failure of anti-virulence drugs and similar damage limitation interventions.

## References

[1] Råberg L, Graham AL, Read AF (2009) Decomposing health: tolerance and resistance to parasites in animals. Philos Trans R Soc Lond B Biol Sci 364: 37–49. 18926971 10.1098/rstb.2008.0184 PMC2666700

[2] Ayres JS, Schneider DS (2011) Tolerance of infections. Annu Rev Immunol 30: 271–294. 10.1146/annurev-immunol-020711-075030 22224770

[3] Medzhitov R, Schneider DS, Soares MP (2012) Disease tolerance as a defense strategy. Science 335: 936–941. 22363001 10.1126/science.1214935 PMC3564547

[4] Gandon S, Mackinnon MJ, Nee S, Read AF (2001) Imperfect vaccines and the evolution of pathogen virulence. Nature 414: 751–756. 11742400 10.1038/414751a

[5] Gandon S, Mackinnon M, Nee S, Read A (2003) Imperfect vaccination: some epidemiological and evolutionary consequences. Proc Biol Sci 270: 1129–1136. 12816650 10.1098/rspb.2003.2370 PMC1691350

[6] Mackinnon MJ, Gandon S, Read AF (2008) Virulence evolution in response to vaccination: the case of malaria. Vaccine 26: C42–C52. 18773536 10.1016/j.vaccine.2008.04.012 PMC2663389

[7] Barclay VC, Sim D, Chan BHK, Nell LA, Rabaa MA, et al (2012) The evolutionary consequences of blood-stage vaccination on the rodent malaria Plasmodium chabaudi. PLoS Biol 10: e1001368 doi:10.1371/journal.pbio.1001368 22870063 10.1371/journal.pbio.1001368 PMC3409122

[8] Gandon S, Day T (2008) Evidences of parasite evolution after vaccination. Vaccine 26: C4–C7. 18773527 10.1016/j.vaccine.2008.02.007

[9] Schneider DS, Ayres JS (2008) Two ways to survive infection: what resistance and tolerance can teach us about treating infectious diseases. Nat Rev Immunol 8: 889–895. 18927577 10.1038/nri2432 PMC4368196

[10] Read AF, Graham AL, Råberg L (2008) Animal defenses against infectious agents: is damage control more important than pathogen control. PLoS Biol 6: e4 doi:10.1371/journal.pbio.1000004 10.1371/journal.pbio.1000004 PMC2605932 19222305

[11] Schneider DS (2011) Tracing personalized health curves during infections. PLoS Biol 9: e1001158 doi:10.1371/journal.pbio.1001158 21957398 10.1371/journal.pbio.1001158 PMC3176750

[12] D'Elia RV, Harrison K, Oyston PC, Lukaszewski RA, Clark GC (2013) Targeting the “Cytokine Storm” for therapeutic benefit. Clin Vaccine Immunol 20: 319–327. 23283640 10.1128/CVI.00636-12 PMC3592351

[13] Barczak AK, Hung DT (2009) Productive steps toward an antimicrobial targeting virulence. Curr Opin Microbiol 12: 490–496. 19631578 10.1016/j.mib.2009.06.012 PMC2763366

[14] Clatworthy AE, Pierson E, Hung DT (2007) Targeting virulence: a new paradigm for antimicrobial therapy. Nat Chem Biol 3: 541–548. 17710100 10.1038/nchembio.2007.24

[15] Rasko DA, Sperandio V (2010) Anti-virulence strategies to combat bacteria-mediated disease. Nat Rev Drug Discov 9: 117–128. 20081869 10.1038/nrd3013

[16] Levin BR, Lipsitch M, Perrot V, Schrag S, Antia R, et al (1997) The population genetics of antibiotic resistance. Clin Infect Dis 24: S9–S16. 8994776 10.1093/clinids/24.supplement_1.s9

[17] Perron GG, Zasloff M, Bell G (2006) Experimental evolution of resistance to an antimicrobial peptide. Proc R Soc B 273: 251–256. 10.1098/rspb.2005.3301 PMC1560030 16555795

[18] MacLean RC, Hall AR, Perron GG, Buckling A (2010) The population genetics of antibiotic resistance: integrating molecular mechanisms and treatment contexts. Nat Rev Genet 11: 405–414. 20479772 10.1038/nrg2778

[19] Little TJ, Allen JE, Babayan SA, Matthews KR, Colegrave N (2012) Harnessing evolutionary biology to combat infectious disease. Nat Med 18: 217–220. 22310693 10.1038/nm.2572 PMC3712261

[20] Janeway C (2004) IMMUNOBIOLOGY 6 PB: the immune system in health and disease. New York: Taylor & Francis. 800 p.

[21] Jamieson AM, Pasman L, Yu S, Gamradt P, Homer RJ, et al (2013) Role of tissue protection in lethal respiratory viral-bacterial coinfection. Science 340: 1230–1234. 23618765 10.1126/science.1233632 PMC3933032

[22] Roy BA, Kirchner JW (2000) Evolutionary dynamics of pathogen resistance and tolerance. Evolution 54: 51–63. 10937183 10.1111/j.0014-3820.2000.tb00007.x

[23] Kover PX, Schaal BA (2002) Genetic variation for disease resistance and tolerance among Arabidopsis thaliana accessions. Proc Natl Acad Sci U S A 99: 11270–11274. 12172004 10.1073/pnas.102288999 PMC123246

[24] Miller MR, White A, Boots M (2006) The evolution of parasites in response to tolerance in their hosts: the good, the bad, and apparent commensalism. Evolution 60: 945–956. 16817535

[25] Råberg L, Sim D, Read AF (2007) Disentangling genetic variation for resistance and tolerance to infectious diseases in animals. Science 318: 812–814. 17975068 10.1126/science.1148526

[26] Best A, White A, Boots M (2008) Maintenance of host variation in tolerance to pathogens and parasites. Proc Natl Acad Sci U S A 105: 20786–20791. 19088200 10.1073/pnas.0809558105 PMC2634923

[27] Best A, White A, Boots M (2010) Resistance is futile but tolerance can explain why parasites do not always castrate their hosts. Evolution 64: 348–357. 19686267 10.1111/j.1558-5646.2009.00819.x

[28] Little TJ, Shuker DM, Colegrave N, Day T, Graham AL (2010) The coevolution of virulence: tolerance in perspective. PLoS Pathog 6: e1001006 doi:10.1371/journal.ppat.1001006 20838464 10.1371/journal.ppat.1001006 PMC2936544

[29] Lefèvre T, Williams AJ, de Roode JC (2011) Genetic variation in resistance, but not tolerance, to a protozoan parasite in the monarch butterfly. Proc Biol Sci 278: 751–759. 20843849 10.1098/rspb.2010.1479 PMC3030843

[30] Vale PF, Wilson AJ, Best A, Boots M, Little TJ (2011) Epidemiological, evolutionary, and coevolutionary implications of context-dependent parasitism. Am Nat 177: 510–521. 21460572 10.1086/659002 PMC3725425

[31] Vale PF, Little TJ (2012) Fecundity compensation and tolerance to a sterilizing pathogen in Daphnia. J Evol Biol 25: 1888–1896. 22856460 10.1111/j.1420-9101.2012.02579.x PMC3798115

[32] Baucom RS, de Roode JC (2011) Ecological immunology and tolerance in plants and animals. Functional Ecology 25: 18–28.

[33] Wu H, Song Z, Hentzer M, Andersen JB, Molin S, et al (2004) Synthetic furanones inhibit quorum-sensing and enhance bacterial clearance in Pseudomonas aeruginosa lung infection in mice. J Antimicrob Chemother 53: 1054–1061. 15117922 10.1093/jac/dkh223

[34] Vilaplana C, Marzo E, Tapia G, Diaz J, Garcia V, et al (2013) Ibuprofen therapy resulted in significantly decreased tissue bacillary loads and increased survival in a new murine experimental model of active tuberculosis. J Infect Dis 208: 199–202. 23564636 10.1093/infdis/jit152

[35] Vale PF, Choisy M, Little TJ (2013) Host nutrition alters the variance in parasite transmission potential. Biol Lett 9: 20121145. 23407498 10.1098/rsbl.2012.1145 PMC3639766

[36] Carey MA, Bradbury JA, Seubert JM, Langenbach R, Zeldin DC, et al (2005) Contrasting Effects of Cyclooxygenase-1 (COX-1) and COX-2 Deficiency on the Host Response to Influenza A Viral Infection. J Immunol 175: 6878–6884. 16272346 10.4049/jimmunol.175.10.6878

[37] Hideko Tatakihara VL, Cecchini R, Borges CL, Malvezi AD, Graça-de Souza VK, et al (2008) Effects of cyclooxygenase inhibitors on parasite burden, anemia and oxidative stress in murine Trypanosoma cruzi infection. FEMS Immunology & Medical Microbiology 52: 47–58. 18031539 10.1111/j.1574-695X.2007.00340.x

[38] Stanley ED, Jackson GG, Panusarn C, Rubenis M, Dirda V (1975) Increased virus shedding with aspirin treatment of rhinovirus infection. JAMA 231: 1248–1251. 163931

[39] Charleston B, Bankowski BM, Gubbins S, Chase-Topping ME, Schley D, et al (2011) Relationship Between Clinical Signs and Transmission of an Infectious Disease and the Implications for Control. Science 332: 726–729. 21551063 10.1126/science.1199884 PMC5844461

[40] Brown SP, Cornforth DM, Mideo N (2012) Evolution of virulence in opportunistic pathogens: generalism, plasticity, and control. Trends in Microbiology 20: 336–342. 22564248 10.1016/j.tim.2012.04.005 PMC3491314

[41] Allen RC, Popat R, Diggle SP, Brown SP (2014) Targeting virulence: can we make “evolution-proof” drugs? Nat Rev Micro In press. 10.1038/nrmicro3232 24625893

[42] Maeda T, García-Contreras R, Pu M, Sheng L, Garcia LR, et al (2012) Quorum quenching quandary: resistance to antivirulence compounds. ISME J 6: 493–501. 21918575 10.1038/ismej.2011.122 PMC3280137

[43] Alizon S, Hurford A, Mideo N, Van Baalen M (2009) Virulence evolution and the trade-off hypothesis: history, current state of affairs and the future. J Evol Biol 22: 245–259. 19196383 10.1111/j.1420-9101.2008.01658.x

[44] Jensen KH, Little TJ, Little T, Skorping A, Ebert D (2006) Empirical support for optimal virulence in a castrating parasite. PLoS Biol 4: e197 doi:10.1371/journal.pbio.0040197 16719563 10.1371/journal.pbio.0040197 PMC1470460

[45] De Roode JC, Altizer S (2010) Host-parasite genetic interactions and virulence-transmission relationships in natural populations of monarch butterflies. Evolution 64: 502–514. 19796153 10.1111/j.1558-5646.2009.00845.x

[46] Rutherford ST, Bassler BL (2012) Bacterial quorum sensing: its role in virulence and possibilities for its control. Cold Spring Harb Perspect Med 2: a012427. 23125205 10.1101/cshperspect.a012427 PMC3543102

[47] O'Loughlin CT, Miller LC, Siryaporn A, Drescher K, Semmelhack MF, et al (2013) A quorum-sensing inhibitor blocks Pseudomonas aeruginosa virulence and biofilm formation. Proc Natl Acad Sci U S A 201316981. 10.1073/pnas.1316981110 PMC3816427 24143808

[48] Tay S, Yew W (2013) Development of quorum-based anti-virulence therapeutics targeting gram-negative bacterial pathogens. Int J Mol Sci 14: 16570–16599. 23939429 10.3390/ijms140816570 PMC3759926

[49] Köhler T, Perron GG, Buckling A, van Delden C (2010) Quorum sensing inhibition selects for virulence and cooperation in Pseudomonas aeruginosa. PLoS Pathog 6: e1000883 doi:10.1371/journal.ppat.1000883 20463812 10.1371/journal.ppat.1000883 PMC2865528

[50] Day T, Graham AL, Read AF (2007) Evolution of parasite virulence when host responses cause disease. Proc R Soc B 274: 2685–2692. 10.1098/rspb.2007.0809 PMC2279213 17711836

[51] Sears BF, Rohr JR, Allen JE, Martin LB (2011) The economy of inflammation: when is less more? Trends Parasitol 27: 382–387. 21680246 10.1016/j.pt.2011.05.004

[52] Joint I, Tait K, Wheeler G (2007) Cross-kingdom signalling: exploitation of bacterial quorum sensing molecules by the green seaweed Ulva. Phil Trans R Soc B 362: 1223–1233. 17360272 10.1098/rstb.2007.2047 PMC2435585

[53] Doeschl-Wilson AB, Kyriazakis I (2012) Should we aim for genetic improvement in host resistance or tolerance to infectious pathogens? Frontiers in Livestock Genomics 272. 10.3389/fgene.2012.00272 PMC3523402 23251138

[54] Doeschl-Wilson AB, Villanueva B, Kyriazakis I (2012) The first step toward genetic selection for host tolerance to infectious pathogens: obtaining the tolerance phenotype through group estimates. Front Gene 3: 265 doi:10.3389/fgene.2012.00265 10.3389/fgene.2012.00265 PMC3571525 23412990

[55] Defoirdt T (2013) Antivirulence therapy for animal production: filling an arsenal with novel weapons for sustainable disease control. PLoS Pathog 9: e1003603 doi:10.1371/journal.ppat.1003603 24130477 10.1371/journal.ppat.1003603 PMC3795005

[56] Gandon S, Mackinnon MJ, Nee S, Read AF (2002) Microbial evolution (Communication arising): Antitoxin vaccines and pathogen virulence. Nature 417: 610–610.

[57] Doeschl-Wilson AB, Bishop SC, Kyriazakis I, Villanueva B (2012) Novel methods for quantifying individual host response to infectious pathogens for genetic analyses. Front Gene 3: 266. 10.3389/fgene.2012.00266 PMC3571862 23413235

[58] Feingold KR, Funk JL, Moser AH, Shigenaga JK, Rapp JH, et al (1995) Role for circulating lipoproteins in protection from endotoxin toxicity. Infect Immun 63: 2041–2046. 7729918 10.1128/iai.63.5.2041-2046.1995 PMC173262

[59] Harris HW, Gosnell JE, Kumwenda ZL (2000) Review: the lipemia of sepsis: triglyceride-rich lipoproteins as agents of innate immunity. J Endotoxin Res 6: 421–430. 11521066

[60] Kantyka T, Plaza K, Koziel J, Florczyk D, Stennicke HR, et al (2011) Inhibition of Staphylococcus aureus cysteine proteases by human serpin potentially limits staphylococcal virulence. Biol Chem 392: 483–489. 21476872 10.1515/BC.2011.044 PMC4372843

[61] Pamplona A, Ferreira A, Balla J, Jeney V, Balla G, et al (2007) Heme oxygenase-1 and carbon monoxide suppress the pathogenesis of experimental cerebral malaria. Nat Med 13: 703–710. 17496899 10.1038/nm1586

[62] Seixas E, Gozzelino R, Chora Â, Ferreira A, Silva G, et al (2009) Heme oxygenase-1 affords protection against noncerebral forms of severe malaria. Proc Natl Acad Sci U S A 106: 15837–15842. 19706490 10.1073/pnas.0903419106 PMC2728109

[63] Ferreira A, Marguti I, Bechmann I, Jeney V, Chora Â, et al (2011) Sickle hemoglobin confers tolerance to Plasmodium infection. Cell 145: 398–409. 21529713 10.1016/j.cell.2011.03.049

[64] Chen F, Liu Z, Wu W, Rozo C, Bowdridge S, et al (2012) An essential role for TH2-type responses in limiting acute tissue damage during experimental helminth infection. Nat Med 18: 260–266. 22245779 10.1038/nm.2628 PMC3274634

[65] Whelton A, Sturmer T, Porter G (2004) Non-steroidal anti-inflammatory drugs. Broe M, Porter G, Bennett W, Verpooten G, editors. Clinical nephrotoxins. Houten: Springer Netherlands. pp. 279–306. Available: http://www.springerlink.com/content/q2v551301mw25r3q/abstract/. Accessed 15 October 2012.

[66] Manefield M, Rasmussen TB, Henzter M, Andersen JB, Steinberg P, et al (2002) Halogenated furanones inhibit quorum sensing through accelerated LuxR turnover. Microbiology 148: 1119–1127. 11932456 10.1099/00221287-148-4-1119

[67] Lesic B, Lépine F, Déziel E, Zhang J, Zhang Q, et al (2007) Inhibitors of pathogen intercellular signals as selective anti-infective compounds. PLoS Pathog 3: e126 doi:10.1371/journal.ppat.0030126 10.1371/journal.ppat.0030126 PMC2323289 17941706

[68] Liu C-I, Liu GY, Song Y, Yin F, Hensler ME, et al (2008) A Cholesterol biosynthesis inhibitor blocks Staphylococcus aureus virulence. Science 319: 1391–1394. 18276850 10.1126/science.1153018 PMC2747771

[69] Baron C (2010) Antivirulence drugs to target bacterial secretion systems. Current Opinion in Microbiology 13: 100–105. 20079679 10.1016/j.mib.2009.12.003

[70] Ng W-L, Perez L, Cong J, Semmelhack MF, Bassler BL (2012) Broad spectrum pro-quorum-sensing molecules as inhibitors of virulence in Vibrios. PLoS Pathog 8: e1002767 doi:10.1371/journal.ppat.1002767 22761573 10.1371/journal.ppat.1002767 PMC3386246

[71] Marozsan AJ, Ma D, Nagashima KA, Kennedy BJ, Kang YK, et al (2012) Protection against Clostridium difficile infection with broadly neutralizing antitoxin monoclonal antibodies. J Infect Dis 206: 706–713. 22732923 10.1093/infdis/jis416 PMC3491748

[72] Bandyopadhaya A, Kesarwani M, Que Y-A, He J, Padfield K, et al (2012) The quorum sensing volatile molecule 2-amino acetophenon modulates host immune responses in a manner that promotes life with unwanted guests. PLoS Pathogens 8: e1003024 doi:10.1371/journal.ppat.1003024 23166496 10.1371/journal.ppat.1003024 PMC3499575

